# Experimental Evaluation of a Concealed Anchoring System for Large-Format Thin Ceramic Panels Under Wind Loading in Ventilated Façades

**DOI:** 10.3390/ma19061062

**Published:** 2026-03-11

**Authors:** Jordi Roviras Miñana, Vicente Sarrablo Moreno, Pedro Casariego Vales

**Affiliations:** LITEIS Research Group (Laboratory of Technological Innovation in Industrialized and Sustainable Edification), School of Architecture, Universitat Internacional de Catalunya (UIC), 08017 Barcelona, Spain

**Keywords:** ventilated façades, large-format ceramic panels, concealed fixing systems, experimental testing, out-of-plane deformation, structural behaviour

## Abstract

Large-format thin ceramic panels are increasingly used in ventilated façade systems due to their reduced weight, high durability and architectural versatility. However, their reduced thickness and large dimensions require reliable anchoring solutions capable of safely transferring wind loads to the supporting structure. This study investigates the structural behaviour of a concealed mechanical anchoring system for large-format porcelain stoneware panels installed in ventilated façades. An experimental campaign was carried out using a full-scale façade prototype representative of real construction conditions. The specimen was subjected to incremental wind pressure and suction loading in a controlled laboratory environment while monitoring the deformation of the ceramic panels, backing support layer and aluminium substructure. The experimental results show that the ceramic panels exhibited stable structural behaviour without cracking or anchor pull-out under pressure levels up to 3006 Pa, exceeding twice the design service pressure. The maximum estimated deflection at the service pressure level (1300 Pa) was 5.7 mm, significantly below the admissible limit defined by the L/200 serviceability criterion. A simplified mechanical analysis based on classical bending theory confirmed that the stresses induced in the ceramic panels remained well below their flexural strength. The results demonstrate that the investigated concealed anchoring system provides reliable structural performance for large-format thin ceramic panels subjected to wind loading in ventilated façade systems, while the simplified analytical verification confirms the mechanical consistency between the measured deformation levels and the flexural capacity of the ceramic material.

## 1. Introduction to Fastening Systems

**Figure 1:**
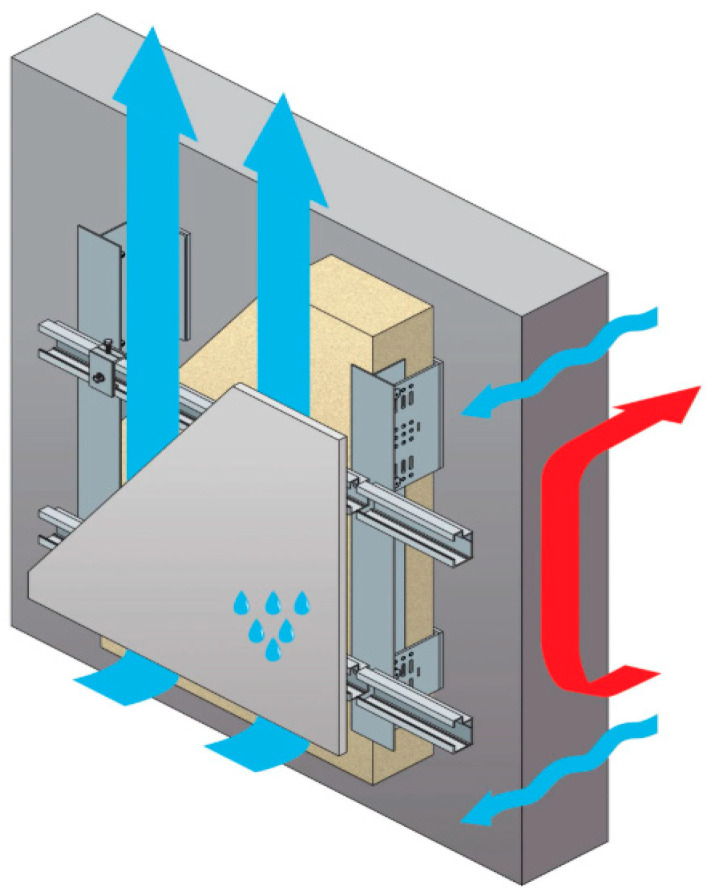
Detail of a ventilated façade system.

Ceramic materials have been used since antiquity as building envelope and cladding elements due to their mechanical resistance, durability and low water absorption capacity [1,2]. Traditional bonded ceramic façade systems have progressively evolved toward mechanically fixed solutions, enabling the installation of larger panel formats and improved structural reliability [3,4]. More recently, the introduction of large-format thin ceramic panels has required further innovation in mechanical anchoring systems, particularly in ventilated façade configurations where the external cladding is structurally separated from the main building envelope [5]. In this regard, it is important to highlight that the ceramic sector has been developing highly advanced technologies applied to ventilated façade solutions for several years [6]. One of the main technological changes that has made it possible to achieve significant progress in this field has been the reduction in the weight of the outer layer, transforming it into a suspended leaf rather than a supported one (Figure 1), where the external cladding is mechanically connected to a supporting substructure separated from the main building enclosure by a ventilated air cavity.

In ventilated façade systems, the external cladding is mounted on a substructure, creating a continuous air cavity between the insulation layer and the outer panel. This configuration contributes to improved hygrothermal behaviour, enhanced durability and protection against environmental exposure, depending on airflow rate and external climatic conditions [7,8]. In addition to thermal performance, ventilated façades provide significant architectural flexibility, allowing diverse material combinations and compositional strategies [9,10].

However, the positive effects of ventilated cavities are strongly influenced by air renewal rate and environmental factors. Previous studies have shown that ventilation performance depends on parameters such as chimney effect, wind-driven flow and pressure losses within the cavity [11].

Among ceramic materials, porcelain stoneware is widely used in façade applications. It is a sintered ceramic material produced from mixtures of clay, quartz and feldspar [12], characterised by very low water absorption (<0.5% according to ISO 13006 [13]), high mechanical strength [14], chemical resistance [15] and dimensional stability [9,16]. These properties derive from its microstructure composed of quartz particles, mullite crystals and a silica-rich amorphous phase with controlled residual porosity [17,18].

Recent technological developments have enabled the production of large-format thin panels (up to 3240 × 1620 mm, thickness 3–12 mm) through advanced sintering and compaction processes [1,19,20,21,22,23,24]. These panels are classified as Group BIa according to EN 14411 [25] and ISO 13006 [13], and their mechanical performance is commonly evaluated according to ISO 10545-4 [26], which defines procedures for determining flexural strength and breaking load. In addition to high bending resistance, sintered porcelain panels exhibit favourable performance under thermal shock, freeze–thaw cycles and UV exposure, making them suitable for exterior façade applications [27]. Flexural strength and breaking load values are strongly dependent on panel thickness and ceramic typology [28,29].

Typical physical and mechanical properties of the porcelain stoneware panels considered in this study are summarised in Table 1. These values correspond to manufacturer-declared properties determined according to standardised testing procedures and are provided as reference material characteristics. The structural wind resistance evaluation presented in this work is based exclusively on the experimental results obtained during the full-scale laboratory testing described in Section 2.

From an environmental perspective, reducing panel thickness contributes to improved sustainability performance through lower raw material consumption, reduced firing energy demand [30], and decreased transportation loads [31]. Life cycle assessment studies indicate that commonly used façade thicknesses between 9 and 12 mm provide favourable environmental performance [32,33,34].

However, reducing the thickness of ceramic panels has direct implications for structural dimensioning, particularly in façade applications where wind-induced bending governs the mechanical response. Although thinner panels enhance environmental efficiency, the decrease in thickness also reduces flexural stiffness, potentially leading to increased deflections under distributed wind loading. In mechanically fixed ventilated façade systems, panels are structurally separated from the primary envelope, and wind pressure and suction generate transverse bending stresses that must remain within the material’s flexural capacity.

To enhance structural reliability and improve post-fracture behaviour, reinforcement systems based on glass fibre mesh bonded to the rear surface using epoxy resins are commonly applied. Experimental investigations have demonstrated that such reinforcement improves ductility and fragment retention capacity under pressure loading conditions, contributing to improved safety performance in façade applications [14,35]. Nevertheless, while reinforcement benefits have been documented, limited research has quantified the relationship between global deformation response under wind loading and the intrinsic flexural strength of large-format thin ceramic panels installed with concealed mechanical anchoring systems.

From a structural engineering perspective, the wind resistance and mechanical behaviour of ventilated façade systems have been investigated through experimental testing and analytical assessment, particularly in curtain walling and mechanically fixed cladding assemblies. Full-scale experimental studies have highlighted the importance of evaluating global façade response under wind loading in order to ensure both serviceability and structural safety [36]. Furthermore, documented façade failures have emphasised the critical role of anchorage configuration and load transfer mechanisms in mechanically fixed systems subjected to environmental actions [37]. Standardised procedures for wind resistance testing of façade systems are established in European regulations and technical assessment documents, such as EN 13116 [38] and EAD 090062-00-0404 [39], which define performance criteria in terms of serviceability and Ultimate Limit States.

Despite the increasing use of large-format thin ceramic panels in ventilated façade systems, the structural behaviour of concealed mechanical anchoring systems under wind loading conditions has not been extensively documented in the scientific literature. Most available studies focus primarily on the mechanical properties of ceramic materials or on small-scale laboratory tests, while relatively limited information is available regarding the full-scale structural response of mechanically anchored façade systems.

In this context, experimental investigations conducted under realistic boundary conditions are essential to better understand the interaction between ceramic panels, anchorage systems and supporting substructures when subjected to wind-induced loads.

The aim of this study is therefore to experimentally evaluate the structural behaviour of a concealed mechanical anchoring system used for the installation of large-format thin porcelain stoneware panels in ventilated façades. A full-scale façade prototype representative of real construction conditions was subjected to incremental wind pressure and suction loading in a controlled laboratory environment.

The experimental results are analysed in terms of pressure–deformation behaviour, serviceability performance and structural response of the anchorage system. In addition, a simplified mechanical model based on classical bending theory is used to verify the consistency between the experimentally observed deformation levels and the theoretical stress state of the ceramic panels.

## 2. Materials and Methods

### 2.1. Cladding System and Specimen Configuration

The experimental campaign was conducted on a full-scale ventilated ceramic façade assembly representative of contemporary industrialised building applications [7,8]. The system consisted of 12 mm thick large-format sintered porcelain stoneware panels mechanically fixed to an aluminium substructure through concealed undercut anchors.

The ceramic panels were commercial ultra-compact sintered porcelain slabs manufactured by Neolith (TheSize Surfaces S.L., Castellón, Spain) [40], classified as Group BIa according to EN 1441 [25] and ISO 13006 [13]. The panels were reinforced on the rear surface with a woven glass fibre mesh (plain weave, taffeta type) bonded using an epoxy resin adhesive. This reinforcement is commonly incorporated in thin large-format panels to enhance post-cracking integrity and improve fragment retention capacity under bending stresses induced by wind loading [35].

**Figure 2:**
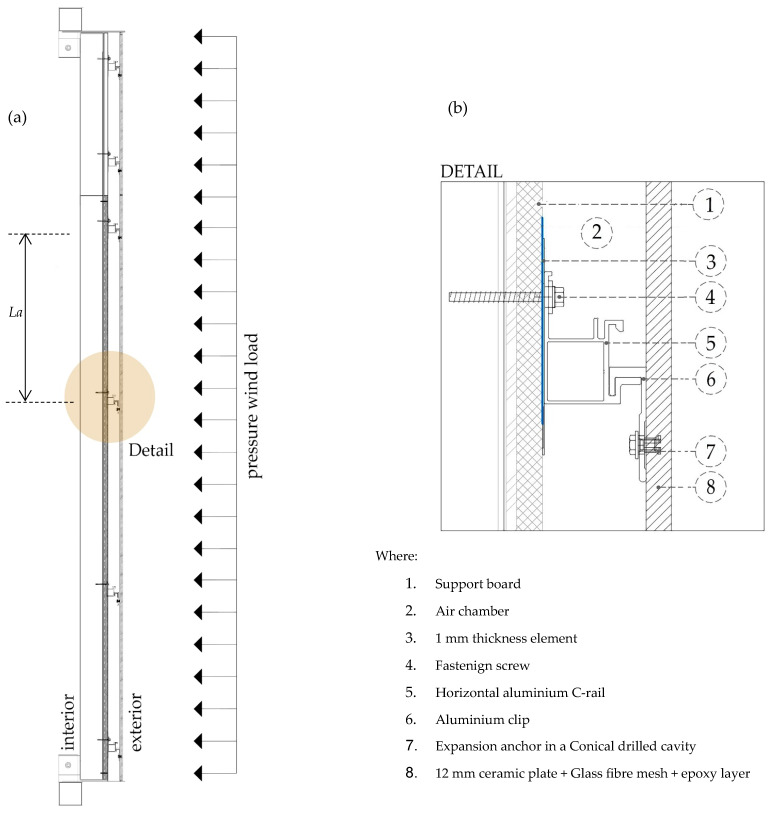
Structural configuration of the tested ventilated façade system: (**a**) vertical section showing load transfer mechanism (Where *L_a_* is the governing anchor spacing in the principal bending direction); (**b**) detail of the concealed undercut anchoring system.

The anchorage system was exclusively mechanical and concealed. It was based on undercut threaded anchors (KEIL Befestigungstechnik GmbH, Engelsbrand, Germany) [41] inserted into precision-drilled blind holes with truncated conical geometry on the rear face of the ceramic panels. The anchors were mechanically connected to horizontal aluminium carrier profiles, which in turn were fixed to vertical aluminium rails anchored to a steel support frame (Figure 2). Such concealed mechanical systems are widely used in ventilated façade solutions due to their structural efficiency [5] and aesthetic advantages [23,42].

The structural configuration of the tested façade assembly and the load transfer mechanism are illustrated in Figure 2.

As illustrated in Figure 2, the ceramic panel behaves as a thin plate supported at discrete anchorage points. Under wind-induced pressure, the load is transferred from the panel to the anchors, then to the aluminium substructure and finally to the supporting frame. For the structural assessment presented in Section 3, two characteristic lengths are distinguished: the panel span $L_{p}$, governing serviceability deflection limits, and the governing anchor spacing $L_{a}$ in the principal bending direction, controlling the bending stress distribution within the ceramic plate.

**Figure 3:**
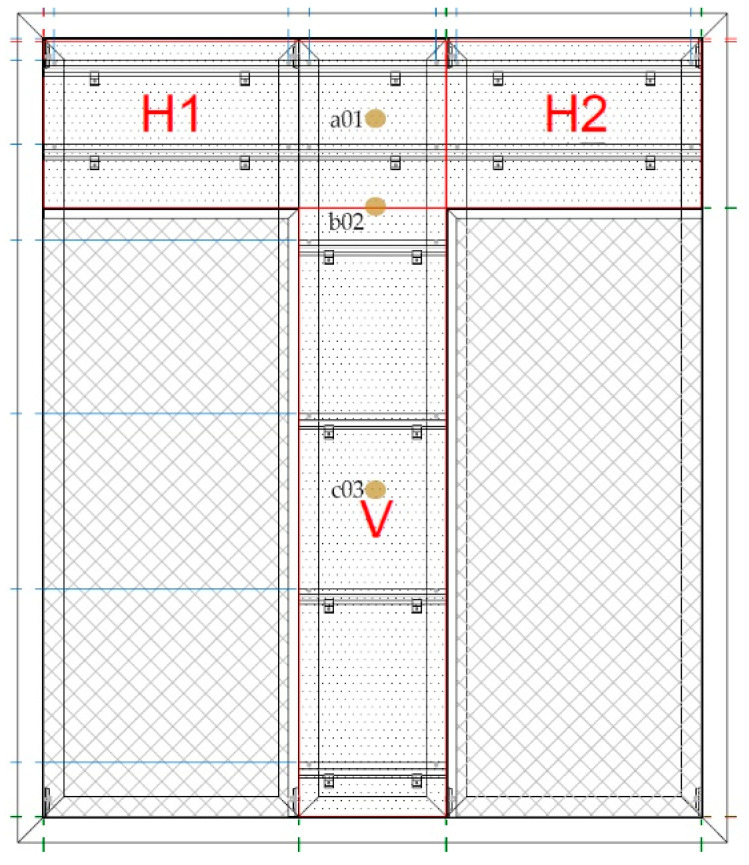
Elevation view of the full-scale façade specimen tested under wind loading (2680 mm × 3200 mm), showing the arrangement of ceramic panel typologies (H1, H2 and V, the distribution of anchorage lines and the location of displacement sensors (a01, b02 and c03).

Three panel typologies were investigated (Figure 3):

- H1 (horizontal panel): 6 anchorage points (61.3 cm horizontal spacing; 34.2 cm vertical spacing).
- H2 (horizontal panel): 4 anchorage points (62.1 cm horizontal spacing; 34.2 cm vertical spacing).
- V (vertical panel): 8 anchorage points (35.91 cm horizontal spacing; 61.1 cm vertical spacing).

These configurations allowed evaluation of the influence of anchor density and orientation on panel deflection behaviour under wind loading.

The complete specimen measured 2680 mm × 3200 mm, resulting in a total exposed surface area of 8576 m^2^. The façade assembly was mounted on a perimeter steel support frame composed of 100 × 100 mm hollow steel sections (2 mm thickness), ensuring rigid boundary conditions during testing.

Behind the ceramic panels, a layered support build-up was installed to replicate realistic façade construction:

- Steel backing plates welded to the structural frame.
- Acoustic membrane layer (5 mm thickness).
- Cement-based support board (12.5 mm thickness).
- Aluminium substructure system.

The panels were fixed exclusively through the concealed anchoring system; no adhesive bonding was used between ceramic and substructure. The configuration reproduced realistic façade installation conditions while ensuring structural continuity across joints and representative load transfer mechanisms.

### 2.2. Design Wind Actions and Regulatory Framework

The design wind actions were defined according to the Spanish Technical Building Code (CTE DB SE-AE [42]) for a building located in the Eixample district (Barcelona), with a façade height below 30 m.

The following parameters were adopted:

- Wind zone: C;
- Basic wind pressure: 0.52 kN/m^2^;
- Terrain category: IV (dense urban environment);
- Exposure coefficient (c_e_): 2.05;
- External pressure coefficients (c_p_): +0.8 (pressure) and −1.2 (suction, corner condition).

From these parameters, the reference pressures used for experimental validation were defined as

- Service pressure (P_1_): 1300 Pa;
- Safety pressure (P_3_): 1950 Pa;
- Partial safety factor: 1.5.

The experimental procedure followed the methodology described in UNE-EN 12179 [43] for wind resistance testing of curtain walling systems and was aligned with the provisions established in ETAG034 [44] and EAD 090062-00-0404 [39] for mechanically fixed external wall cladding kits.

Serviceability and Ultimate Limit State criteria were defined as follows:

- Serviceability Limit State (SLS):

Maximum mid-span deflection limited to *L_p_*/200 under service pressure. For a characteristic span of 3000 mm, the allowable deflection was therefore 15 mm. A characteristic span of 3000 mm was adopted, corresponding to the nominal length of the largest ceramic slab configuration typically used in large-format ventilated façade applications.

- Ultimate Limit State (ULS):

No rupture of ceramic panels, no anchor pull-out or failure, and no permanent deformation of structural profiles under safety pressure.

### 2.3. Full-Scale Experimental Setup and Instrumentation

The experimental campaign was conducted in a certified façade testing laboratory using a full-scale pressure chamber designed for wind load resistance assessment. The specimen was mounted on a rigid steel reaction frame and connected to a sealed pressurisation chamber capable of applying both positive pressure and negative pressure (suction) in a controlled manner.

#### 2.3.1. Test Chamber and Boundary Conditions

The façade assembly (2680 mm × 3200 mm) was installed in a sealed pressure chamber to simulate wind-induced pressure differentials. Perimeter sealing profiles were incorporated, and panel joints were temporarily sealed during testing to ensure airtightness and proper pressure development, in accordance with standard wind testing procedures.

The supporting steel frame (100 × 100 mm hollow sections, 2 mm thickness) provided rigid boundary conditions, minimising global frame deformation and isolating the structural response of the façade system.

Environmental conditions during testing were maintained within laboratory-controlled ranges:

- Temperature: 20 °C;
- Relative humidity: 75%;
- Atmospheric pressure: 101.3 kPa.

These parameters were recorded prior to testing to ensure consistency with standardised procedures.

#### 2.3.2. Pressure Generation and Control System

Wind pressure and suction were generated using a mechanically controlled turbine system integrated into the test chamber. The equipment allowed automated switching between positive and negative pressure, enabling simulation of wind pressure cycles.

Pressure was applied incrementally in predefined load steps with real-time monitoring. The pressure measurement system had a maximum uncertainty of ±5%, as specified in the laboratory calibration documentation. Measurement uncertainty was expressed with a coverage factor k = 2.

#### 2.3.3. Displacement Measurement and Data Acquisition

The structural response of the façade assembly was monitored using six linear displacement transducers LVDTs (HBM GmbH, Darmstadt, Germany) with a minimum measurement resolution of 0.1 mm, connected to a digital data acquisition system. The transducers were connected to a digital data acquisition system recording pressure and displacement simultaneously.

Sensor distribution was defined to capture both global and local behaviour:

- a01, b02, c03: positioned on ceramic panels to measure panel deflection.
- a04 and c06: positioned on the cement-based support board layer.
- b05: positioned on the vertical aluminium profile.

**Figure 4:**
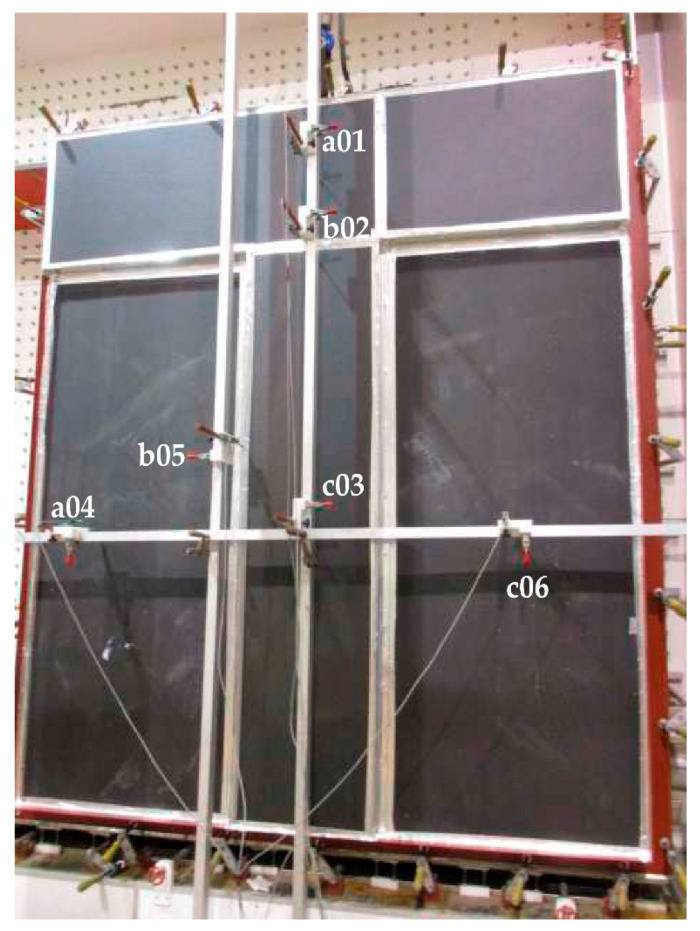
Photograph of the full-scale façade prototype installed in the wind pressure test chamber, indicating the position of the displacement transducers (LVDT sensors a01–c06) used to monitor the deformation of the ceramic panels, support board and aluminium substructure during the experimental campaign.

Sensor c03 was placed at the geometric centre of the main ceramic panel to measure maximum mid-span deflection. The exact location of the displacement transducers installed on the façade prototype is shown in Figure 4.

Net panel deflection was obtained by subtracting support displacements from central displacement values, thereby eliminating rigid-body movements and isolating bending deformation of the ceramic component. Pressure and displacement data were recorded continuously during each load step, including both instantaneous peak deformation and residual deformation measured after load removal.

The measurement instrumentation used during the experimental campaign was calibrated according to the laboratory quality procedures. The main measurement devices and their associated uncertainties are summarised in Table 2. The expanded measurement uncertainty was expressed as the standard measurement uncertainty multiplied by a coverage factor k = 2, corresponding to an approximate confidence level of 95% assuming a normal distribution of measurement errors.

The expanded measurement uncertainty was expressed as the standard measurement uncertainty multiplied by a coverage factor k=2, corresponding to an approximate confidence level of 95% assuming a normal distribution of measurement errors.

#### 2.3.4. Loading Protocol

The loading sequence consisted of incremental pressure steps applied as follows:

- Two initial pulses at 300 Pa;
- One pulse at 500 Pa;
- One pulse at 1000 Pa;
- Subsequent increments of 200 Pa.

Each load step had a duration of 10 s. After each pressure or suction stage, residual deformation was recorded one minute after load removal. After the final pressure stage, residual deformation was recorded again after one hour.

Four test phases were performed on the same specimen:

- Pressure test up to 2000 Pa;
- Suction test up to 2400 Pa;
- Repetition of pressure test up to 3000 Pa;
- Repetition of suction test up to failure condition.

The test was terminated when structural instability occurred in the support board layer during suction at approximately 3275 Pa.

The experimental campaign was conducted on a single full-scale prototype representative of the façade configuration. Due to the scale and complexity of the assembly, statistical repetition of specimens was not performed. However, the loading protocol included repeated pressure and suction cycles, allowing evaluation of the consistency of the structural response under progressive loading conditions.

## 3. Results and Structural Assessment

### 3.1. Experimental Load-Deflection Behaviour

The wind resistance tests were performed under incremental pressure and suction loading according to the protocol described in Section 2. The structural response of the façade assembly was monitored through six displacement sensors positioned on the ceramic panels, support board and aluminium substructure.

**Figure 5:**
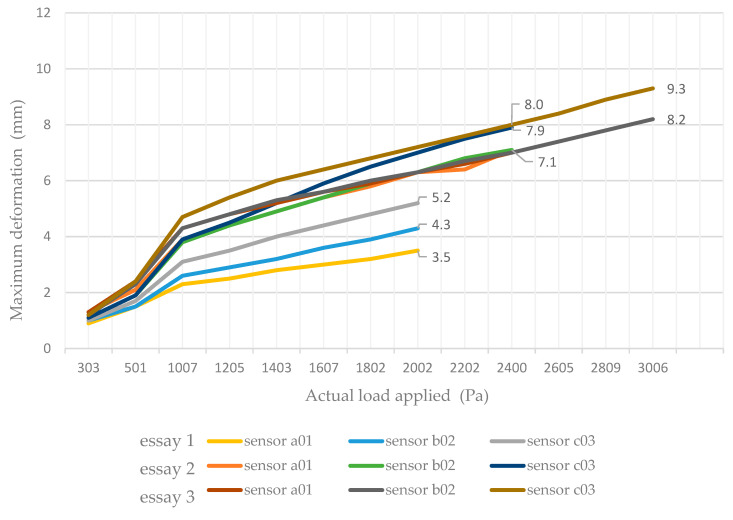
Relationship between applied wind pressure and maximum out-of-plane deformation measured by the ceramic panel sensors (a01, b02 and c03) during the three experimental test sequences.

Figure 5 shows the relationship between the applied wind pressure and the maximum out-of-plane deformation measured by the displacement sensors installed on the ceramic panels (a01, b02 and c03) during the three experimental tests performed on the prototype. Consequently, the figure includes nine deformation curves, corresponding to the measurements recorded by the three sensors in each test sequence. The curves show a similar deformation trend for the three sensors, with increasing deformation as wind pressure increases, indicating a consistent structural response of the ceramic panel across the different test sequences.

Among the ceramic sensors, c03, located at the geometric centre of the panel, represents the mid-span position and is therefore considered the most representative sensor for evaluating the global bending behaviour of the ceramic panel under wind loading.

The maximum displacement recorded at the central ceramic sensor was

$$\delta_{max}=9.3\;mm$$

at an applied pressure of

$$q_{max}=3006\;Pa$$

The pressure–deflection curve exhibits an approximately linear behaviour up to approximately 2400 Pa, followed by a moderate increase in deformation slope at higher pressure levels. No brittle cracking or anchor detachment was observed in the ceramic panel during the pressure phase. The three ceramic sensors (a01, b02 and c03) exhibited consistent deformation trends, confirming that the measured behaviour was representative of the global panel response of the ceramic panels under wind loading. Table 3 summarises the maximum deflection values recorded at each sensor location.

### 3.2. Structural Behaviour Under Pressure and Suction

Under positive pressure, the ceramic panels exhibited progressive deformation without visible cracking, anchor pull-out or permanent deformation of the aluminium substructure. The deformation response remained stable throughout the loading sequence, indicating that the concealed anchorage system provided adequate load transfer between the ceramic cladding and the supporting substructure.

Under suction loading, larger displacement values were recorded in the backing support board layer (sensors a04 and c06). It should be noted, however, that in the laboratory test configuration this support layer was directly exposed to the pressure differential generated in the test chamber. In real ventilated façade systems, the backing board is normally protected by the external ceramic cladding and does not directly receive wind pressure.

Consequently, the relatively large displacements measured in the support board should be interpreted as part of the experimental boundary conditions rather than as a governing structural limitation of the façade system.

More importantly, the ceramic panels and their concealed anchorage system maintained structural integrity throughout the test sequence. No ceramic cracking, anchor pull-out or local damage was observed, even at pressure levels significantly exceeding the design service pressure.

The test sequence was terminated at approximately 3275 Pa due to instability of the backing support layer under suction loading.

### 3.3. Serviceability Limit State (SLS) Verification

The design service pressure defined for the project was

$$q_{\mathrm{service}\;}=1300\;Pa$$

This value was derived from the wind load calculations performed for the reference building location according to the Spanish Technical Building Code (CTE DB-SE-AE [42]), considering the exposure conditions described in Section 2.

The serviceability criterion limits the maximum mid-span deflection of the ceramic panel to

(1) $$\delta_{adm}=\frac{L_{p}}{200}$$

which corresponds to commonly adopted façade serviceability criteria used in wind resistance assessment of façade systems and curtain walling (e.g., EN 13116 [38] and façade engineering practice).

For a characteristic span of

$$L_{p}=3000\;mm$$

the admissible deflection becomes

(2) $$\delta_{adm}=\frac{3000}{200}=15\;mm$$

Experimental measurements obtained at sensor c03, located at the geometric centre of the ceramic panel, indicate the following deformation values in the vicinity of the service pressure:

1205 Pa→5.4 mm

1403 Pa→6.0 mm

Assuming linear interpolation between these load levels, the estimated deflection at service pressure is

$$\delta_{1300}\approx 5.7\;mm$$

The ratio between measured service deflection and allowable deflection is

(3) $$\frac{\delta_{1300}}{\delta_{adm}}=\frac{5.7}{15}\approx 0.38$$

This result indicates that the façade system satisfies the SLS requirements with a significant margin relative to the allowable deformation limit. This margin indicates that the façade system operates well within the serviceability deformation limits under the design wind pressure.

### 3.4. Simplified Mechanical Modelling

To evaluate whether the experimentally observed deformation levels are mechanically consistent with the flexural capacity of the ceramic material, a simplified analytical verification was performed. The objective of this verification is not to reproduce the full structural complexity of the façade assembly—characterised by semi-rigid anchors, substructure flexibility and backing layer deformability—but rather to provide an order-of-magnitude assessment of the stress levels induced in the ceramic panel under wind pressure.

#### 3.4.1. Structural Idealisation

Based on the discrete support layout described in Section 2, the ceramic panel can be conservatively idealised as a strip spanning the governing anchor spacing $L_{a}$ in the principal bending direction and subjected to a uniformly distributed pressure *q*.

This simplified representation is commonly adopted in the mechanical assessment of mechanically anchored façade panels when evaluating the bending stresses between anchorage points using classical Euler–Bernoulli beam theory [45].

For a simply supported strip under uniform pressure, the maximum bending moment is as follows [45]:

(4) $$M_{max}=\frac{qL_{a}^{2}}{8}$$

The maximum bending stress in a rectangular section is given by

(5) $$\sigma =\frac{My}{I}$$

For a plate of thickness *h* the maximum bending stress becomes

(6) $$\sigma_{max}=\frac{6M_{max}}{bh^{2}}$$

Substituting the expression for $M_{max}$ and simplifying leads to

(7) $$\sigma_{max}=0.75\frac{qL_{a}^{2}}{h^{2}}$$

#### 3.4.2. Simplified Bending Stress Check

Using the maximum applied pressure (*q*) and the governing anchor spacing ($L_{a}$) with panel thickness (*h*)

$$\begin{array}{ll} & q=3006\;Pa\\ & L_{a}=0.621\;m\\ & h=0.012\;m \end{array}$$

the maximum bending stress becomes

(8) $$\sigma_{max}=0.75\times 3006\times \frac{(0.621{)}^{2}}{(0.012{)}^{2}}$$

which results in:

$$\sigma_{max}\approx 6\;Mpa$$

The average flexural strength of the tested porcelain stoneware panels exceeds

MOR>35 Mpa

Since

$$\sigma_{max}\ll MOR$$

The induced stress under maximum applied wind pressure remains significantly below the material flexural capacity.

This simplified check is consistent with the absence of global ceramic panel fracture during testing.

#### 3.4.3. Analytical–Experimental Consistency

The simplified mechanical model predicts stress levels well within the elastic capacity of the ceramic material. The experimentally measured maximum deflection of 9.3 mm occurred at more than twice the service pressure and did not produce cracking or anchorage failure.

Differences between the simplified analytical prediction and the measured deformation response can be attributed to several factors, including:

- Semi-rigid behaviour of concealed anchors;
- Local rotational flexibility at anchorage points;
- Deformation of the aluminium substructure;
- Interaction between the ceramic panel and the backing support layers.

Nevertheless, the order of magnitude of the analytical stress estimation is consistent with the experimentally observed behaviour.

### 3.5. Ultimate Limit State (ULS) Assessment

The safety pressure adopted for the façade system was defined as

$$q_{s\mathrm{afety}\;}=1950\;Pa$$

This value corresponds to the design wind pressure multiplied by a partial safety factor, in accordance with the design criteria described in Section 2.

During the experimental campaign, the maximum applied pressure reached

$$q_{\max\;}=3006\;Pa$$

which corresponds to approximately

$$q_{\max\;}=2.31q_{service}$$

At the safety pressure level, no ceramic cracking, anchor pull-out or permanent deformation of the aluminium profiles was observed.

Even at the maximum applied pressure of 3006 Pa, the ceramic panels maintained structural integrity and the concealed anchorage system remained mechanically stable.

The experimental sequence was ultimately terminated due to instability of the backing support layer under suction loading rather than failure of the ceramic panel or the anchorage system.

These results indicate that the ceramic cladding system satisfies the ultimate limit state requirements under wind loading conditions exceeding the design safety pressure.

## 4. Conclusions

This study presented an experimental full-scale evaluation of a concealed mechanical anchoring system for large-format, thin porcelain stoneware panels used in ventilated façade applications. The investigation combined laboratory wind pressure testing with a simplified mechanical consistency assessment in order to validate the structural behaviour of the ceramic component.

Based on the experimental results and analytical verification, the following conclusions can be drawn:

- Structural response under wind loading. The ceramic panels exhibited stable and progressive deformation under incremental pressure loading up to 3006 Pa. The maximum mid-span displacement recorded at the central sensor was 9.3 mm, without cracking, anchor pull-out or brittle fracture of the ceramic material.
- Serviceability Limit State (SLS) compliance. Under the design service pressure of 1300 Pa, the estimated mid-span deflection was approximately 5.7 mm. This value represents only 38% of the admissible serviceability limit defined by L/200 (15 mm for a 3000 mm span), demonstrating that the façade system satisfies serviceability requirements with a significant safety margin.
- Mechanical consistency of experimental results. A simplified analytical verification based on classical beam theory was performed to estimate the bending stresses in the ceramic panels under wind loading. The comparison between calculated stress levels and the flexural strength of the porcelain stoneware material establishes a direct relationship between global panel deformation and intrinsic material resistance, providing a mechanical interpretation of the experimental behaviour. The estimated maximum bending stress under the highest applied pressure was approximately 6 MPa, which remains well below the average flexural strength of the porcelain stoneware material (MOR > 35 MPa). This confirms that the experimental load levels are theoretically consistent with the absence of global panel fracture.
- Ultimate limit state (ULS) behaviour. At the defined safety pressure of 1950 Pa, no structural damage was observed in the ceramic panel, anchoring system or aluminium substructure. Even at the maximum applied pressure (3006 Pa), corresponding to more than twice the service pressure, structural integrity of the ceramic component was maintained. The governing instability was associated with the backing support layer under suction rather than with the ceramic cladding itself.
- Contribution to façade engineering practice. The results provide experimental evidence supporting the structural viability of concealed mechanical anchoring systems for large-format thin ceramic panels. The combination of full-scale testing and analytical verification contributes to bridging the gap between laboratory evaluation and real façade design practice.

Overall, the study demonstrates that the investigated system achieves adequate structural performance under both service and safety wind load conditions, confirming its suitability for ventilated façade applications in urban environments.

Although the experimental campaign was conducted on a single full-scale specimen, the repeated loading cycles provided consistent deformation trends, supporting the reliability of the obtained results.

## Figures and Tables

**Table 1:** Reference physical and mechanical properties of the tested porcelain stoneware panels (manufacturer data according to standardised procedures).

Property	Standard	Declared Value	Unit
Water absorption	ISO 13006 [13]	<0.1	%
Flexural strength (MOR)	ISO 10545-4 [26]	>35	MPa
Breaking load	ISO 10545-4 [26]	>1300	N
Thickness	-	12	mm
Density	ISO 10545-3 [26]	~2400	kg/m^3^

**Table 2:** Measurement instrumentation and associated uncertainty used during the experimental campaign.

Parameter Measured	Instrumentation	Measurement Uncertainty
Displacement	Linear displacement transducers (LVDT)	Resolution 0.1 mm
Differential pressure	Pressure control system of the wind test chamber	±5%
Temperature	Laboratory environmental sensor	±3 °C
Relative humidity	Laboratory environmental sensor	±5%
Atmospheric pressure	Barometric pressure sensor	±1 kPa
Linear measurements (installation)	Calibrated measuring tape	±1 mm

**Table 3:** Maximum measured deflections per sensor and loading phase.

Test 1 (Maximum real load = 2002 Pa)		
**Element**	**Sensor**	**Maximum deflection** (mm)
Ceramic plate	a01	3.5
	b02	4.3
	c03	5.2
Support panel (Aquapanel)	a04	20.6
	c06	30.4
Substructure (profile)	b05	13.1
Test 2 (Maximum real load = 2400 Pa)		
**Element**	**Sensor**	**Maximum deflection** (mm)
Ceramic plate	a01	7.1
	b02	7.1
	c03	7.9
Support panel (Aquapanel)	a04	40.4
	c06	60.1
Substructure (profile)	b05	24.6
Test 3–4 (Maximum real load = 3006 Pa)		
**Element**	**Sensor**	**Maximum deflection** (mm)
Ceramic plate	a01	8.2
	b02	8.2
	c03	9.3
Support panel (Aquapanel)	a04	54.1
	c06	73.9

## Data Availability

The original contributions presented in this study are included in the article. Further inquiries can be directed to the corresponding author.
